# The Isoquinoline-Sulfonamide Compound H-1337 Attenuates SU5416/Hypoxia-Induced Pulmonary Arterial Hypertension in Rats

**DOI:** 10.3390/cells11010066

**Published:** 2021-12-27

**Authors:** Hiroki Shoji, Yoko Yoshida, Takayuki Jujo Sanada, Akira Naito, Junko Maruyama, Erquan Zhang, Kengo Sumi, Seiichiro Sakao, Kazuo Maruyama, Hiroyoshi Hidaka, Koichiro Tatsumi

**Affiliations:** 1 Department of Respirology, Graduate School of Medicine, Chiba University, Chiba 260-8670, Japan; zugzwang_h_spitz@yahoo.co.jp (H.S.); akira_n_med-respi@yahoo.co.jp (A.N.); sakaos@faculty.chiba-u.jp (S.S.); tatsumi@faculty.chiba-u.jp (K.T.); 2 Department of Respiratory Medicine, Tokyo Rosai Hospital, Tokyo 143-0013, Japan; 3 D. Western Therapeutics Institute, Inc., Nagoya 460-0003, Japan; yoyoshida@dwti.co.jp (Y.Y.); kesumi@dwti.co.jp (K.S.); hihidaka@dwti.co.jp (H.H.); 4 Human Research Promotion and Drug Development, Mie University, Mie 514-8507, Japan; 5 Department of Anesthesiology and Critical Care Medicine, School of Medicine, Mie University, Mie 514-8507, Japan; j-maru@suzuka-u.ac.jp (J.M.); zhangequan@hotmail.com (E.Z.); k-maru@clin.medic.mie-u.ac.jp (K.M.); 6 Faculty of Medical Engineering, Suzuka University of Medical Science, Mie 510-0293, Japan

**Keywords:** pulmonary hypertension, Rho-associated protein kinase signaling, mammalian target of rapamycin signaling, animal model of pulmonary arterial hypertension, right ventricular remodeling

## Abstract

Pulmonary arterial hypertension (PAH) is characterized by elevated pulmonary arterial pressure and right heart failure. Selective pulmonary vasodilators have improved the prognosis of PAH; however, they are not able to reverse pulmonary vascular remodeling. Therefore, a search for new treatment agents is required. H-1337 is an isoquinoline-sulfonamide compound that inhibits multiple serine/threonine kinases, including Rho-associated protein kinase (ROCK) and mammalian target of rapamycin (mTOR). Here, we investigated the effects of H-1337 on pulmonary hypertension and remodeling in the pulmonary vasculature and right ventricle in experimental PAH induced by SU5416 and hypoxia exposure. H-1337 and H-1337M1 exerted inhibitory effects on ROCK and Akt. H-1337 inhibited the phosphorylation of myosin light chain and mTOR and suppressed the proliferation of smooth muscle cells in vitro. H-1337 treatment also suppressed the phosphorylation of myosin light chain and mTOR in the pulmonary vasculature and decreased right ventricular systolic pressure and the extent of occlusive pulmonary vascular lesions. Furthermore, H-1337 suppressed aggravation of right ventricle hypertrophy. In conclusion, our data demonstrated that inhibition of ROCK and mTOR pathways with H-1337 suppressed the progression of pulmonary vascular remodeling, pulmonary hypertension, and right ventricular remodeling.

## 1. Introduction

Pulmonary arterial hypertension (PAH) is characterized by sustained elevation of pulmonary artery pressure and progressive obstructive changes in the pulmonary arteries, which leads to subsequent right heart failure and high mortality [1]. Since the 1990s, selective pulmonary vasodilators targeting endothelin, nitric oxide, and prostacyclin pathways have been developed [2], and advances in treatments, including combination therapies with selective pulmonary vasodilators, have contributed to improved survival rates in patients with PAH [3]. However, PAH is still incurable [1] and patients with PAH who exhibit inadequate responses despite medical treatment require lung transplantation [4]. The incurability of PAH results from pulmonary vascular remodeling, which cannot be reversed by pulmonary selective vasodilators [5,6]. Pulmonary vascular remodeling, which is a pathological feature of PAH, occludes the pulmonary arteries and induces pulmonary hypertension [1]. Within the remodeled pulmonary vessels, abnormal proliferation of pulmonary artery endothelial cells and smooth muscle cells (SMCs) [7] with anti-apoptotic features has been observed [8,9]. Thus, to identify improved curative treatments for PAH, other approaches targeting reversal of complex vascular lesions are required [1].

The Rho-associated protein kinase (ROCK) pathway is associated with the development of PAH. Activation of ROCK by vascular stimulants, such as hypoxia and endothelin-1 (ET-1), activates myosin light chain (MLC) via direct phosphorylation of MLC and inactivation of myosin light chain phosphatase (MLCP) [10,11]. Sustained vasoconstriction decreases the vascular bed and increases shear stress and is associated with the development of pulmonary vascular remodeling and PAH [1,10,12]. The expression of Rho A and ROCK is increased and MLCP is inactivated in the lungs of PAH animal models induced by hypoxia or monocrotaline (MCT) treatment [13,14,15,16]. The activated ROCK pathway has also been reported in patients with PAH [17].

Similarly, the mammalian target of rapamycin (mTOR) pathway has also been shown to be associated with PAH development. The activated mTOR pathway induces the proliferation of SMCs [18,19] and is associated with the development of vascular remodeling in PAH [20]. Moreover, the mTOR pathway is activated in experimental PAH animal models induced by hypoxia, MCT, and Su5416/hypoxia (Su/Hx) rat models, which are associated with the development of pulmonary vascular remodeling and right ventricle (RV) remodeling and dysfunction [21,22,23] Thus, activation of the ROCK and Akt/mTOR pathways plays important roles in PAH development, and inhibition of these pathways may be a new strategy for treating PAH.

H-1337 is an isoquinoline-sulfonamide compound first synthesized by D. Western Therapeutics Institute, Inc. (Nagoya, Japan) in 2010. We have reported that H-1337 and H-1337M1 can inhibit ROCK1/2 and other serine/threonine kinases [24,25]. Additionally, H-1337 and its metabolite H-1337M1 were found to have inhibitory activities on Akt in a preliminary experiment. Although inhibition of ROCK or Akt pathways has been shown to suppress the development of PAH [20,26], the effects of inhibition of both ROCK and Akt pathways on PAH have not been investigated. Thus, we hypothesized that H-1337 may exert antiproliferative effects on vascular lesion cells and pulmonary vasodilative effects via inhibition of both ROCK and Akt signaling.

Accordingly, the purpose of this study was to evaluate the effects of H-1337 on hemodynamics and vascular remodeling in Su/Hx rats and assess the mechanism involved. In this study, H-1337 and H-1337M1 were characterized, and the effects of H-1337 and H-1337M1 on SMCs and Su/Hx rats were evaluated.

## 2. Materials and Methods

Details of protocols used in this study are shown in the supplemental methods.

### 2.1. Reagents and Antibodies

H-1337 was supplied by D. Western Therapeutics Institute, Inc. (Nagoya, Japan). Details of other reagents and antibodies are provided in the supplemental material.

### 2.2. Cell Proliferation Analyses

Human pulmonary artery SMCs (hPASMCs) were cultured in Humedia-SG2 (Kurabo, Osaka, Japan) until stimulation and were plated in 96-well plates at 2.5 × 10^3^ cells/well in serum-starved medium (Humedia-SB supplemented with 1% fetal bovine serum [FBS] and antibiotics [Kurabo]). Then, hPASMCs were stimulated with platelet-derived growth factor (PDGF)-BB and incubated with H-1337, H-1337M1, LY294002, or Fasudil for 72 h. The proliferation of hPASMCs was evaluated using a Cell Counting Kit-8 (Dojindo Laboratories, Kumamoto, Japan).

### 2.3. Western Blot Analyses

Before stimulation, 2 × 10^5^ hPASMCs were cultured with serum-starved medium (Humedia-SB containing 1% FBS and antibiotics [Kurabo]), followed by stimulation with serum-starved medium containing 0.1 µM ET-1 or 10 ng/mL recombinant PDGF and treatment with different reagents. The isolated proteins were separated and analyzed by western blotting.

### 2.4. Design of Animal Experiments

Five-week-old male Sprague-Dawley rats were divided into the following three groups: (1) Su/Hx + H-1337 group, Su/Hx rats administered H-1337, (2) Su/Hx + Vehicle group, Su/Hx rats without administration of H-1337, and (3) control group without any treatment. The Su/Hx model was prepared as described in our previous report [27]. Right heart catheterization, right ventricular assessment, and histological analysis were performed for all rats at 5 weeks.

### 2.5. Preparing Su5416/Hypoxia Model

The Su/Hx model was established as described in our previous report [27]. The rats were treated with a single injection of SU5416 (20 mg/kg) and a 3-week exposure to hypoxia (10% O_2_), which was followed by normoxia exposure for 2 weeks. The Su/Hx + H-1337 group was continuously treated with H-1337 from day 0 to day 35.

### 2.6. Treatments with H-1337 for Su/Hx Rats

H-1337 was dissolved in drinking water at 0.25 mg/mL for the Su/Hx + H-1337 group. The Su/Hx + H-1337 group was continuously treated with H-1337 from day 0 to day 35. H-1337 solution was changed every 2 days. The details of the validation and data from preliminary experiments are shown in the supplemental methods.

### 2.7. Histological Assessment of Pulmonary Vascular Remodeling

Lung sections were stained with Elastica van Gieson staining to evaluate the extent of pulmonary vascular remodeling. The arteries were analyzed under a microscope (Nikon ECLIPSE 55i; Nikon, Tokyo, Japan) and were scored based on the severity of luminal occlusion and the distribution of α-smooth muscle actin (α-SMA)-positive cells, as follows: no evidence of neointimal formation (grade 0), partial (<50%) luminal occlusion (grade 1), and severe (>50%) luminal occlusion (grade 2) according to a previously described method with minor modifications [27,28]. RV myocyte hypertrophy and RV fibrosis were evaluated as previously described, with slight modifications [29,30].

### 2.8. Immunofluorescence, Immunohistochemistry, Kinase Assays, and Measurement of Serum Concentrations of H-1337 and H-1337M1

The details of the protocols used are given in the supplemental methods.

### 2.9. Statistical Analyses

Quantitative data are presented as means ± standard deviations unless otherwise stated. Comparisons between two or three or more groups were made using Student’s *t*-test or analysis of variance (ANOVA) with Bonferroni’s post-hoc test, respectively. Statistical significance was set at *p* < 0.05. Statistical analyses were performed using the GraphPad Prism software program, version 8.0.2 (GraphPad Software, La Jolla, CA, USA).

## 3. Results

### 3.1. Characteristics of H-1337 and the Metabolite H-1337M1

**Figure 1:**
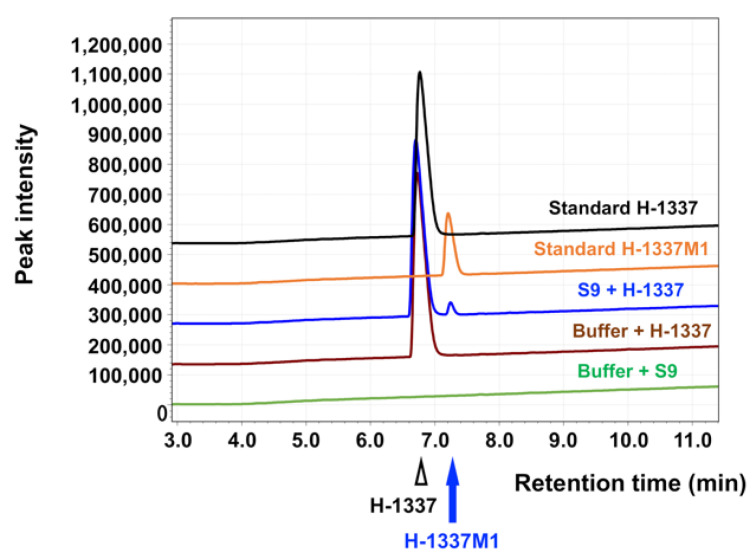
Analysis of H-1337M1 using high-performance liquid chromatography. H-1337 was incubated with rabbit liver S9 fraction. A peak distinct from that of H-1337 was detected (blue). The metabolite was named as H-1337M1 by the authors.

First, H-1337 and its metabolite H-1337M1 were pharmacologically characterized. H-1337 can be metabolized by liver enzymes to H-1337M1 (Figure 1). Importantly, H-1337 and H-1337M1 exerted inhibitory effects on ROCK and Akt (Table 1).

### 3.2. H-1337 and H-1337M1 Suppressed the Phosphorylation of MLC in Human SMCs

**Figure 2:**
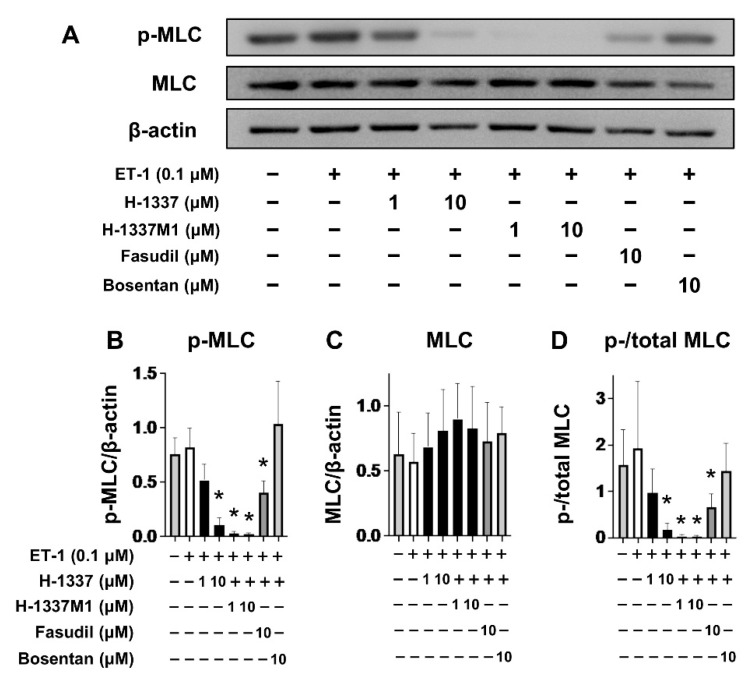
H-1337 and H-1337M1 suppressed the phosphorylation of myosin light chain (MLC) in human pulmonary artery smooth muscle cells (hPASMCs). hPASMCs were treated with H-1337, H-1337M1, Fasudil (a ROCK inhibitor), or bosentan (a specific endothelin receptor antagonist) for 1 h, followed by stimulation with endothelin-1 (ET-1; 0.1 µM) for 1 h. (**A**) Representative images of western blot analysis. (**B**,**C**) Quantification of western blots for phosphorylated MLC (p-MLC) and total MLC. (**D**) The ratio of band intensity of p-MLC to total MLC. *: analyzed by ANOVA, *p* < 0.05 versus groups treated with ET-1 without H-1337, H-1337M1, fasudil, or bosentan.

Next, the effects of H-1337 and H-1337M1 on the phosphorylation of MLC in hPASMCs were assessed by western blotting (Figure 2A). H-1337 at 10 μM and H-1337M1 at 1 μM and 10 μM significantly decreased the phosphorylation of MLC similar to Fasudil (a ROCK inhibitor) at 10 μM (Figure 2B,D). Bosentan, a specific endothelin receptor antagonist, decreased the phosphorylation of MLC (Figure 2D), although the difference did not reach significant levels. These results suggested that H-1337 and H-1337M1 exert inhibitory effects on the phosphorylation of MLC in hPASMCs. Additionally, myosin light chain kinase (MLCK) was assessed using the kinase assay. The assay suggested that 0.1 μM H-1337 M1 suppressed 76% of the kinase activity of MLCK, while the inhibition rates of ROCK-1 and -2 were 95 and 87%, respectively (Table S1). The expression and phosphorylation of myosin phosphatase targeting subunit 1 (MYPT1) was also analyzed using western blotting. However, ET-1 stimulation did not increase the phosphorylation of MYPT1 at Thr696, which is involved in the inhibition of MLCP activity in humans [31]. Therefore, evaluating the effect of H-1337, H-1337M1, and Fasudil on MYPT-1 was difficult (Figure S2).

### 3.3. H-1337 and H-1337M1 Suppressed the Phosphorylation of mTOR in Human SMCs

**Figure 3:**
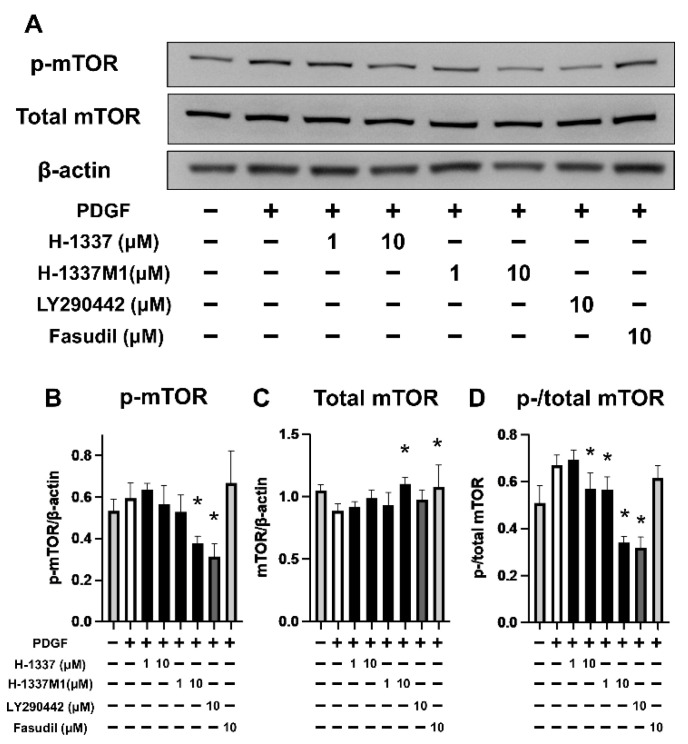
Phosphorylation of the Akt/mammalian target of rapamycin (mTOR) pathway in human pulmonary artery smooth muscle cells (hPASMCs). hPASMCs were treated with H-1337, H-1337M1, fasudil (ROCK inhibitor), or LY294002 (phosphatidylinositol 3-kinase inhibitor) for 1 h, followed by stimulation with platelet-derived growth factor (PDGF; 10 ng/mL) for 1 h. (**A**) Representative photographs of western blots. (**B**–**D**) Quantification of the data from (**A**). (**B**) Mammalian target of rapamycin (mTOR). (**B**) Phosphorylated mTOR (p-mTOR). (**C**) mTOR. (**D**) The ratio of phospho- to total mTOR. *: analyzed by ANOVA, *p* < 0.05 versus groups treated with PDGF without H-1337, H-1337M1, fasudil, or LY294002.

Next, the effect of H-1337 on mTOR in hPASMCs were assessed (Figure 3). Western blotting suggested that H-1337 at 1 µM and H-1337M1 at 1 µM and 10 µM significantly reduced the phosphorylation of mTOR induced by PGDF (Figure 3D) as well as LY294002, which is an inhibitor of phosphatidylinositol 3-kinase (PI3K) that is known to block mTOR activation [32]. Conversely, fasudil, a ROCK inhibitor, did not have a suppressive effect on the phosphorylation of mTOR (Figure 3B,D). These results suggested that H-1337 and H-1337M1 exert a suppressive effect on the phosphorylation of mTOR in hPASMCs.

### 3.4. H-1337 and H-1337M1 Suppressed the Proliferation of hPASMCs

**Figure 4:**
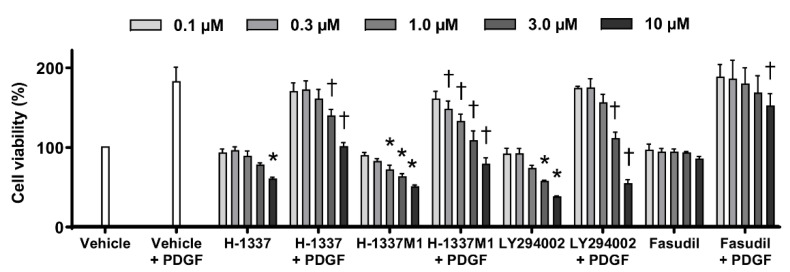
Antiproliferative effects in human pulmonary artery smooth muscle cells (hPASMCs). The viability of hPASMCs treated with H-1337, H-1337M1, and LY294002 was assessed using Cell Counting Kit-8 assays. hPASMCs were incubated with platelet-derived growth factor (PDGF; 10 ng/mL) and treated with different concentrations (0.1–10 µM) of H-1337, H-1337M1, LY294002 (phosphatidylinositol 3-kinase inhibitor), or fasudil (ROCK inhibitor). *: analyzed by ANOVA, *p* < 0.05 versus vehicle; †: *p* < 0.05 versus vehicle + PDGF.

To assess the effects of H-1337 on the proliferation of PASMCs, cell proliferation assays were performed. H-1337, H-1337M1, and LY294002 suppressed the proliferation of hPASMCs induced by stimulation with PDGF in a concentration-dependent manner (Figure 4). Conversely, a significant suppressive effect of fasudil was observed only at the highest concentration (10 μM).

### 3.5. H-1337 Decreased Right Ventricular Pressure and Occlusive Vascular Lesions in Su/Hx Rats

Before evaluating the effect of H-1337 on Su/Hx rats, the experimental condition was optimized (Table S2 and Figure S1). The details are described in the Supplemental methods. Based on the optimization, H-1337 was diluted with drinking water to a concentration of 0.25 mg/mL and was continuously administered to Su/Hx rats for 5 weeks.

**Figure 5:**
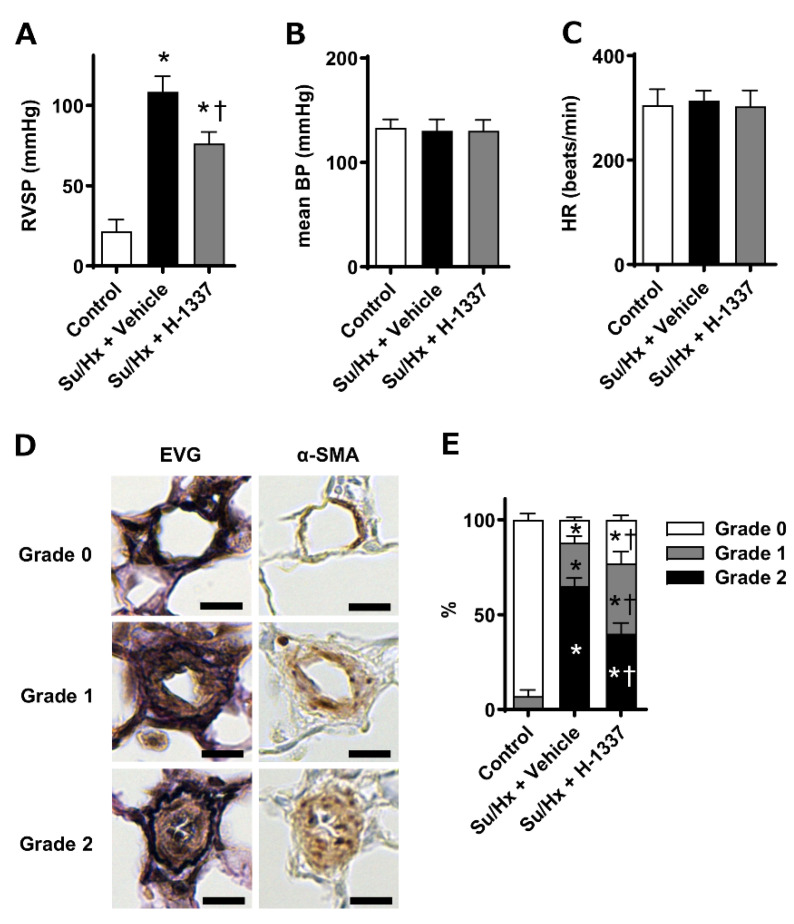
Suppressive effects of H-1337 on the aggravation of hemodynamics and vascular remodeling in SU5416/hypoxia (Su/Hx) rats. Hemodynamic and pathological assessment for rats were conducted at 5 weeks. (**A**) Right ventricular systolic pressure (RVSP). (**B**) Mean blood pressure (mBP). (**C**) Heart rate (HR). (**D**) Representative photomicrographs of pulmonary arteries stained with Elastica van Gieson (EVG) and immunostained with α-smooth muscle actin (α-SMA) Scale bars indicate 10 μm. (**E**) Quantified data from pulmonary vascular remodeling. Su/Hx + H-1337: H-1337-treated Su/Hx rats (*n* = 7); Su/Hx + vehicle: vehicle-treated Su/Hx rats (*n* = 6); and control: untreated rats (*n* = 8). *: analyzed by ANOVA, *p* < 0.05 versus the control; †: *p* < 0.05 versus Su/Hx + vehicle.

Next, hemodynamics and vascular remodeling were assessed at 5 weeks. The right ventricular systolic pressure (RVSP) values of the control, Su/Hx + vehicle, and Su/Hx + H-1337 groups were 21.7 ± 7.2 mmHg, 108.4 ± 9.9, and 76.4 ± 7.1 mmHg, respectively (*n* = 6–8). The RVSP value of the Su/Hx + H-1337 group was significantly lower than that of the Su/Hx + vehicle group (Figure 5A), whereas no significant differences in mean blood pressure and heart rate were observed among the three groups (Figure 5B,C). The extent of pulmonary vascular remodeling was histologically assessed. The percentages of obstructive lesions (grade 2) in the Su/Hx + H-1337 group were significantly lower than those in the Su/Hx + vehicle group (Figure 5D,E). These results suggested that the H-1337 treatment suppressed the progression of pulmonary vascular remodeling and the elevation of RVSP in Su/Hx rats.

### 3.6. H-1337 Suppressed the Phosphorylation of MLC and mTOR in the Pulmonary Vasculature

**Figure 6:**
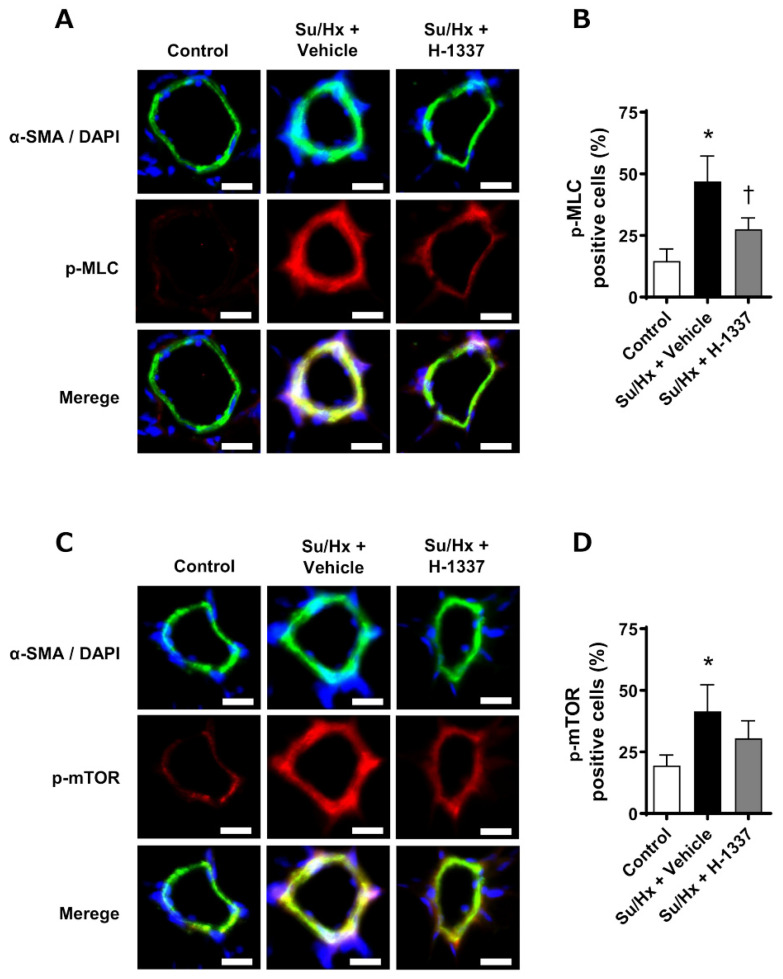
Phosphorylation of MLC and mTOR was suppressed by H-1337 in pulmonary arteries. (**A**) Immunofluorescence of α-smooth muscle actin (α-SMA, green) and phospho-myosin light chain (MLC, red) in pulmonary arteries. Nuclei were counterstained with DAPI (blue). (**B**) Quantification of the immunofluorescence of phosphorylated MLC (p-MLC). (**C**) Representative images of immunofluorescence of α-SMA (green) and phospho-mammalian target of rapamycin (mTOR, red) in pulmonary arteries. Nuclei were counterstained with DAPI (blue). (**D**) Quantification of the immunofluorescence of phospho-mTOR. Su/Hx + H-1337: H-1337-treated Su/Hx rats (*n* = 3); Su/Hx + vehicle: vehicle-treated Su/Hx rats (*n* = 3); and control: untreated rats (*n* = 3). All scale bars indicate 10 μm. *: analyzed by ANOVA, *p* < 0.05 versus the control; †: *p* < 0.05 versus the Su/Hx + vehicle group.

To evaluate the local effects of H-1337 on the pulmonary arteries, immunofluorescence was performed (Figure 6). The percentages of phospho-MLC-positive to α-SMA-positive cells in the control, Su/Hx + vehicle, and Su/Hx + H-1337 groups were 14.7% ± 4.9%, 47.0% ± 10.2%, and 27.6% ± 4.5%, respectively (Figure 6B). The percentages of phospho-mTOR-positive to α-SMA-positive cells in the control, Su/Hx + vehicle, and Su/Hx + H-1337 groups were 19.5% ± 4.3%, 41.5% ± 10.8%, and 30.6% ± 7.1%, respectively (Figure 6D). Thus, H-1337 treatment significantly decreased the phosphorylation of MLC and mTOR in pulmonary vasculature in Su/Hx rats.

### 3.7. H-1337 Had a Suppressive Effect on RV Remodeling

**Figure 7:**
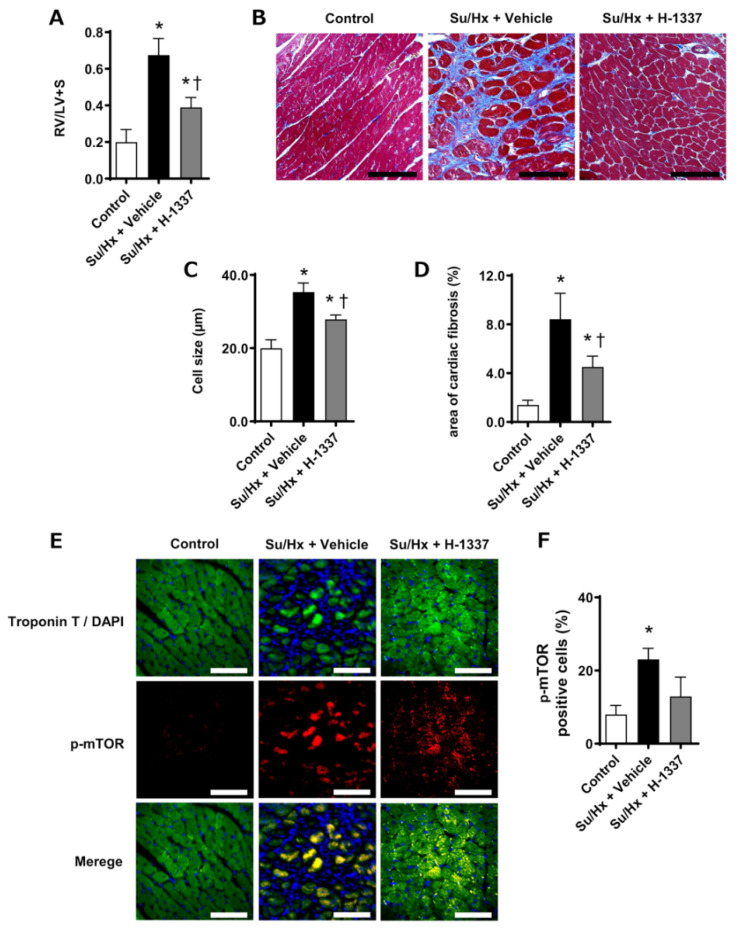
Suppressive effects of H-1337 on right ventricle remodeling in SU5416/hypoxia (Su/Hx) rats. (**A**) The weight ratio of the right ventricle to the left ventricle + septum (RV/LV+S) in Su/Hx rats at 5 weeks. (**B**) Histological images of right ventricle stained with Masson’s trichrome. Scale bars indicate 100 μm. (**C**) Quantification of the size of cardiomyocytes. (**D**) Quantified data of the area of cardiac fibrosis. (**E**) Immunofluorescence of the right ventricle for troponin T and phospho-mammalian target of rapamycin (mTOR). Scale bars indicate 50 μm. (**F**) The proportion of phospho-mTOR (p-mTOR)-positive to troponin T-positive cells (cardiomyocytes) in Su/Hx rats at 5 weeks. For hemodynamic and histological assessments, control: untreated rats (*n* = 8); Su/Hx + vehicle: vehicle-treated Su/Hx rats (*n* = 6); and Su/Hx + H-1337: H-1337-treated Su/Hx rats (*n* = 7). For immunofluorescence, control: (*n* = 3); Su/Hx + vehicle: (*n* = 3); and Su/Hx + H-1337 (*n* = 3). All scale bars indicate 10 μm. *: analyzed by ANOVA, *p* < 0.05 versus the control; †: *p* < 0.05 versus the Su/Hx + vehicle group.

Finally, the effects of H-1337 on RV hypertrophy and remodeling were examined. The RV/LV+S ratios in the control, Su/Hx + vehicle, and Su/Hx + H-1337 groups were 0.20 ± 0.07, 0.68 ± 0.09, and 0.39 ± 0.06, respectively (*n* = 6–8). The RV/LV+S ratio in the Su/Hx + H-1337 group was significantly lower than that in the Su/Hx + vehicle group (Figure 7A). Masson’s trichrome staining (Figure 7B) revealed that the sizes of cardiomyocytes and the fibrotic area of the RV were significantly decreased in the Su/Hx + H-1337 group compared with those in the Su/Hx + vehicle group (Figure 7D). Immunofluorescence revealed that H-1337 decreased phosphorylation of mTOR in Su/Hx rats (Figure 7E,F). These results suggested that H-1337 treatment suppressed the phosphorylation of mTOR in cardiomyocytes and RV remodeling in Su/Hx rats.

## 4. Discussion

In the current study, the effects of the multiple serine/threonine kinase inhibitor H-1337 on Su/Hx rats were assessed. H-1337 suppressed the phosphorylation of MLC and mTOR and the proliferation of hPASMCs. The results of animal experiments with Su/Hx rats suggested that H-1337 administration suppressed the phosphorylation of MLC and mTOR in the SMCs of pulmonary arteries and attenuated pulmonary hypertension and pulmonary vascular remodeling. H-1337 also suppressed RV hypertrophy and fibrotic changes, accompanied by the suppression of mTOR phosphorylation in cardiomyocytes.

H-1337 and H-1337M1 were characterized in this study. It was demonstrated that that H-1337 and H-1337M1 exerted inhibitory effects on ROCK and Akt in vitro. Previously, we demonstrated that H-1337 exerted inhibitory effects on several kinases including ROCK [24]. In the previous report, IC50 values of Fasudil for ROCK-1 and ROCK-2 inhibition were over 1 and 0.73 μM, respectively, which were higher than those of H-1337 and H-1337M1. The inhibitory potential of H-1337 and H-1337M1 on ROCK-1 and ROCK-2 appeared to be potentially higher than fasudil. Akt is a serine/threonine kinase that is involved in the activation of mTOR via phosphorylation of proline-rich Akt/PKB substrate 40 kDa and tuberous sclerosis complex 2 [18,19,33,34]. In the present study, inhibition of Akt by H-1337 and H-1337 M1 was newly demonstrated.

It was found that H-1337 can be metabolized by liver enzymes to H-1337M1. There exists a possibility that H-1337 is metabolized by not only the liver but also the kidney, although the renal metabolism of H-1337 was not evaluated in this study. It is necessary to investigate the detailed drug disposition of H-1337 and H-1337M1 in future studies.

H-1337 and H-1337M1 suppressed MLC phosphorylation in cultured PASMCs. Phosphorylation of MLC can be induced by three methods: (1) phosphorylation by ROCK; (2) phosphorylation by MLCK; and (3) inactivation of MYPT1 by ROCK [10]. MYPT1 is a component of the MLCP complex, and phosphorylation of MYPT1 induces the suppression of MLCP activity [10,35]. In the present study, western blotting revealed a clear suppressive effect of H-1337 and H-1337M1 on phosphorylation of MLC. However, the kinase assay suggested a moderate suppressive effect of H-1337M1 on MLCK. Thus, it was presumed that H-1337 and H-1337M1 could suppress ROCK and MLCK, which may induce the phosphorylation of MLC. Conversely, the effects of H-1337 and H-1337M1 on MYPT-1 could not be evaluated in this study, as ET-1 did not promote the phosphorylation of MYPT1 at Thr696. Woodsome et al. have reported that ET-1 stimulation did not alter the level of phosphorylation of MYPT1 at Thr696 in SMCs [36]. Thr696 is considered to maintain a high ratio of phosphorylation even at resting condition, which may be the cause of the lack of response to ET-1 [36]. In future studies, to evaluate the effect of H-1337 on MYPT1, it may be helpful to use other agonists such as calyculin A, which enhance the phosphorylation of Thr696 [36].

Phosphorylation of mTOR and proliferation of cultured PASMC were suppressed by H-1337 and H-1337M1. The activated mTOR pathway is related to the proliferation of SMCs [18,19]. LY294002 suppresses the activation of mTOR pathway via inhibition of PI3K [32] and the proliferation of SMCs [37]. Houssaini et al. have reported that the proliferation of PASMCs derived from MCT-induced PAH rats was suppressed by rapamycin [22], an allosteric inhibitor of mTOR complex 1 [18]. Notably, restoring the mTOR pathway by upregulating phosphatase and tensin homolog, a regulator of mTOR [20], induces apoptosis in PASMCs [38]. In the present study, 10 μM H-1337M1 suppressed the activation of mTOR and the cell viability of hPASMCs at a level similar to that of LY294002, although the effects of 1 and 10 μM H-1337 and 1 μM H-1337 M1 on mTOR were moderate. It has been known that activated MLC is also responsible for the proliferation of PASMCs [26]. Therefore, inhibition of both the MLC pathway with H-1337 and H-1337M1 might be related to the suppression of hPASMC proliferation.

The phosphorylation of MLC in the pulmonary vasculature was suppressed by the H-1337 treatment in Su/Hx rats. Increased vascular tone is a major contributor to increased pulmonary arterial pressure [1,39]. Akt pathway regulates the vascular tone via the activation of ROCK and phosphorylation of MLC [10]. Suppression of MLC phosphorylation via inhibition of Akt can relax the constriction of PASMCs [14]. Several ROCK inhibitors can suppress the elevation RVSP and the development of vascular remodeling in experimental PAH models induced by hypoxic exposure or MCT treatment [26,40,41], which supports our results. Thus, the inhibition of the Akt pathway by H-1337 treatment may be associated with decreased RVSP in Su/Hx rats.

H-1337 treatment attenuated the activated mTOR pathway in the pulmonary vasculature and pulmonary vascular remodeling in Su/Hx rats. It has been known that the mTOR pathway is activated in the lungs of Su/Hx rats [23] and MCT [22] and hypoxia-induced PH rats [21], which supported our results. Suppression of mTOR can attenuate pulmonary vascular remodeling. mTOR inhibition has been reported to decrease the extent of pulmonary vascular remodeling in Su/Hx animal models [23,27], and hypoxia-induced and MCT models [16,22,42,43]. These results support our current findings. Our previous study revealed that the severity of pulmonary vascular remodeling was closely correlated with the elevation of RVSP in Su/Hx rats [44]. It could be inferred from these results that suppression of mTOR using H-1337 may contribute to the attenuation of pulmonary vascular remodeling and the decrease of pulmonary arterial pressure in Su/Hx rats.

Notably, H-1337 also had a suppressive effect on RV hypertrophy and fibrosis in Su/Hx rats. Crosstalk occurs between cardiomyocytes and the extracellular matrix via the mTOR pathway, which cooperatively regulates myocardial hypertrophy and fibrosis [45,46]. Moreover, the Akt/mTOR pathway is activated in RV with PAH, and inhibition of mTOR suppresses RV hypertrophy and fibrosis and improves RV function [23]. However, it is still unclear whether the suppression of RV hypertrophy and fibrosis was an indirect effect associated with improved pulmonary hemodynamics or a direct effect of H-1337 on RV. Thus, further studies are needed to fully elucidate the effects of H-1337 on RV function.

The current study had several limitations. First, the treatment effects of H-1337 were demonstrated only in Su/Hx PAH rats, which are thought to mimic human PAH but not fully recapitulate human PAH. Second, in this study, we focused on prevention of PAH development; therefore, it is unclear whether H-1337 is effective for the treatment of PAH. Additionally, signaling pathways other than the ROCK and mTOR pathways were not investigated in this study. Signals from these other pathways may affect the ROCK and/or mTOR pathways and therefore the results of our study. Finally, the side effects of H-1337 were not fully investigated in this study. Fasudil has been reported to cause renal impairment [47], and H-1337 may cause similar adverse effects. In future studies, the side effects of H-1337 should be investigated. Despite these limitations, we believe that dual inhibition of the Rho and Akt pathways with H-1337 could suppress the aggravation of PAH.

In conclusion, H-1337 and H-1337M1 exert inhibitory effects on the ROCK and mTOR pathways in hPASMC and the pulmonary vasculature in Su/Hx rats and may contribute to attenuation of pulmonary hypertension and remodeling of the pulmonary vasculature and RV in Su/Hx rats.

## Figures and Tables

**Table 1:** Kinase inhibition profiles of H-1337 and H-1337M1.

Kinase	H-1337 IC_50_ (µM)	H-1337M1 IC_50_ (µM)
ROCK1	0.24	0.02
ROCK2	0.32	0.012
Akt1	0.279	0.0042
Akt2	1.662	0.054
Akt3	0.112	0.0253

## Data Availability

The data from this study are available upon request from the corresponding author.

## Supplementary Materials

The following supporting information can be downloaded at: https://www.mdpi.com/article/10.3390/cells11010066/s1, Table S1: Kinase inhibition rate under the treatment with 0.1 μM H-1337M1, Table S2: Stability of H-1337 in water at 50 °C, Figure S1: Optimization of H-1337 treatment in rats, Figure S2: The expression of myosin phosphatase targeting subunit 1 (MYPT1) and the phosphorylated MYPT-1 (p-MYPT1) in human pulmonary artery smooth muscle cells (hPASMCs), Supplemental methods.
