# Supplementary material for: The Isoquinoline-Sulfonamide Compound H-1337 Attenuates SU5416/Hypoxia-Induced Pulmonary Arterial Hypertension in Rats
Source: Cells. 2021 Dec 27;11(1):66. doi: 10.3390/cells11010066 (PMC8750965; doi:10.3390/cells11010066)
Supplement: Supplementary file 1 [file cells-11-00066-s001.zip › cells-1375945-supplementary.pdf]

cells

MDPI

The Isoquinoline-Sulfonamide Compound H-1337 Attenuates SU5416/Hypoxia-Induced Pulmonary Arterial Hypertension in Rats

1. Supplemental Data

Table S1. Kinase inhibition rate under treatment with 0.1 uM H-1337M1.

Kinases	Inhibition rate (%)
ROCK1	95
ROCK2	87
MLCK	76

Table S2. Stability of H-1337 in water at 50 °C.

Time (days)	0	3	7	14	28
Content	99.71%	99.71%	99.70%	99.74%	99.72%
Residual Rate (%)	100%	100%	99.9%	100%	100%

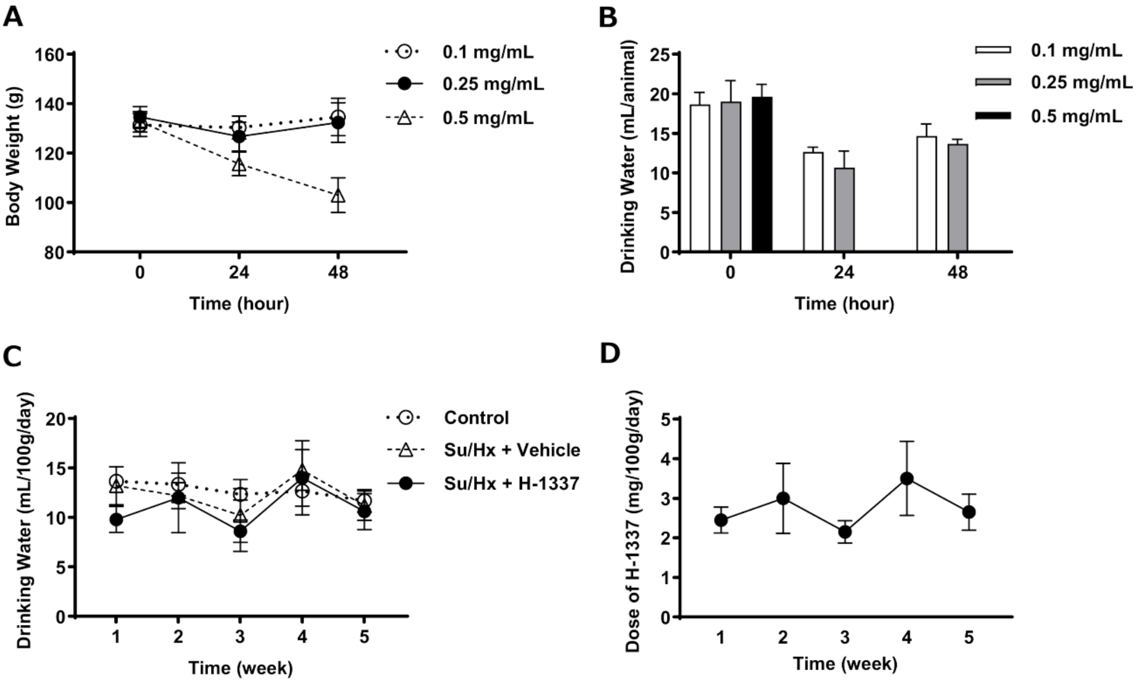

Figure S1. Optimization of H-1337 treatment in rats. H-1337 was dissolved in drinking water at concentrations of 0.1, 0.25, or 0.5 mg/mL (**A** and **B**), and the solution was administered to Sprague-Dawley rats. (**A**) Time-course changes in body weight. (**B**) Time-course changes in water intake. (**C**) Water intake under the conditions adopted in this study (H-1337 solution: 0.25 mg/mL). (**D**) Dose of H-1337 per animal calculated from water intake.

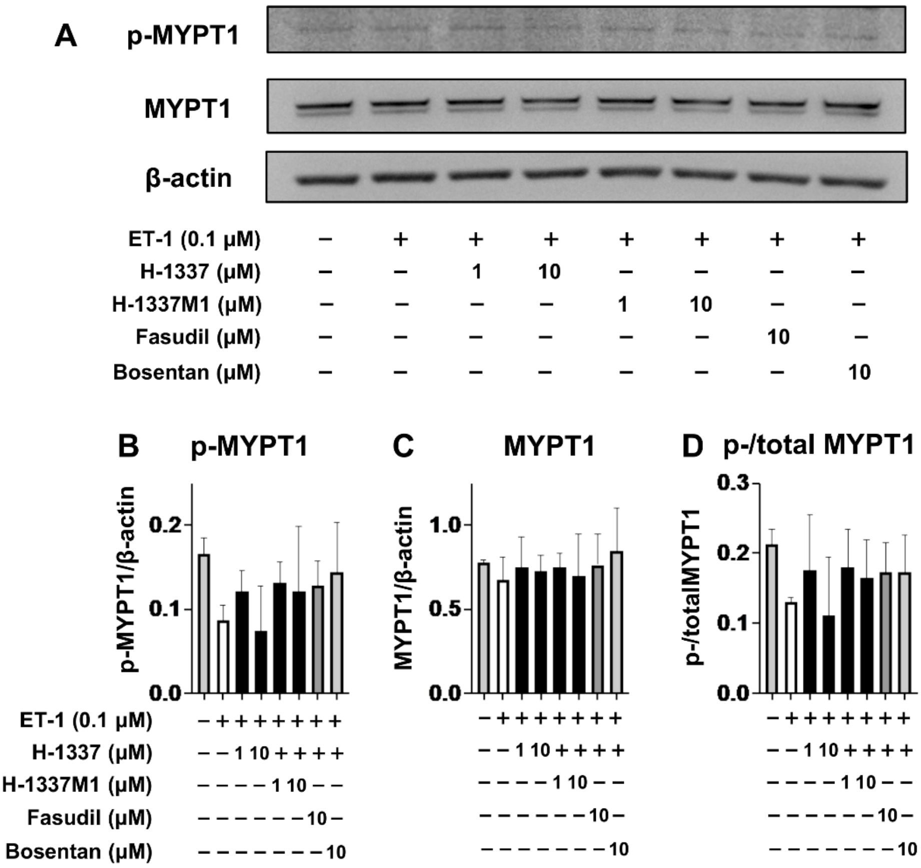

Figure S2. The expression of myosin phosphatase targeting subunit 1 (MYPT1) and the phosphorylated MYPT1 (p-MYPT1) in human pulmonary artery smooth muscle cells (hPASMCs).

hPASMCs were treated with H-1337, H-1337M1, fasudil (a ROCK inhibitor), or bosentan (a specific endothelin receptor antagonist) for 1 hour, followed by stimulation with endothelin-1 (ET-1; 0.1 μM) for 1 hour. After treatment, protein was extracted and subjected to western blot analysis. (**A**) Representative images of western blot analysis. (**B** and **C**) Quantification of the western blots for p-MYPT1 and MYPT1. (**D**) The ratio of the band intensity of p-MYPT1 to that of total MYPT1. The difference between the groups treated with ET-1 without H-1337 and the other groups were analyzed using ANOVA. All of the differences were insignificant.

2. Supplemental Methods

2.1. Ethical Statements

This study was conducted in accordance with the guidelines of the Declaration of Helsinki and approved by the Review Board for Animal Experiments of Chiba University (approval numbers 29-445, 30-126, and 1-83) and the Institutional Animal Research Committee of the Mie University (approval number 21-41). The study was performed in accordance with the guidelines of the Animal Research Committee of Laboratory Animal Center, Graduate School of Medicine, Chiba University and the Institutional Animal Research Committee of the Mie University.

2.2. Reagents and Antibodies

H-1337 was supplied by D.Western Therapeutics Institute (Nagoya, Japan). Fasudil was purchased from LC Laboratories (Woburn, MA, USA). Bosentan was purchased from Tokyo Chemical Industry (Tokyo, Japan). LY294002 was purchased from Chemdea (Rockwood, NJ, USA). The following antibodies were used for immunohistochemistry: anti-α-SMA (cat. no. ab7817; Abcam, Cambridge, UK), anti-cardiac troponin T (1/200; cat. no. ab8295; Abcam), anti-phospho-MLC (cat. no. 95777; Cell Signaling Technology, Danvers, MA, USA), anti-phospho-mTOR (cat. no. 109268; Abcam), anti-mouse IgG conjugated with horseradish peroxidase (cat. no. E0433; Dako, Santa Clara, CA, USA), anti-mouse IgG Alexa Fluor Plus 488 (cat. no. A32723; Invitrogen, Carlsbad, CA, USA), and anti-rabbit IgG Alexa Fluor Plus 594 (cat. no. A32740; Invitrogen). The following antibodies were used for western blot analysis: anti-MLC (cat. no. 3672; Cell Signaling Technology), anti-phospho-MLC (cat. no. 3674; Cell Signaling Technology), anti-myosin phosphatase targeting subunit 1 (MYPT1; cat. no. 8574; Cell Signaling Technology), anti-phospho-MYPT1 (Thr696; cat. no. 5163; Cell Signaling Technology), anti-mTOR (cat. no. 2983; Cell Signaling Technology), anti-phospho-mTOR (cat. no. 5536; Cell Signaling Technology), anti-β-actin (cat. no. 622102; BioLegend, San Diego, CA, USA), and anti-rabbit IgG conjugated with horseradish peroxidase (cat. no. W401B; Promega, Madison, WI, USA).

2.3. Cell Culture

hPASMCs were purchased from Kurabo (Osaka, Japan). The cells were cultured in 6-cm Petri dishes coated with fibronectin (Corning, Corning, NY, USA) and Humedia-SG2 (Kurabo) at 37 °C and 5% CO_2 in a humidified air incubator until the other assays were performed.

2.4. Cell Proliferation Analysis

hPASMCs were cultured in Humedia-SG2 (Kurabo) until stimulation and were plated in 96-well plates at 2.5×10^3 cells/well in serum-starved medium (Humedia-SB supplemented with 1% FBS and antibiotics [Kurabo]). Then, hPASMCs were incubated with 10 ng/mL recombinant PDGF-BB (PeproTech, Rocky Hill, NJ, USA) and 0–10 μM H-1337, 0–10 μM H-1337M1, 0–10 μM LY294002 (Ridgewood, NJ, USA), or 0–10 μM Fasudil (LC Laboratories) for 72 h. The proliferation of hPASMCs was evaluated using Cell Counting Kit-8 assays (Dojindo Laboratories, Kumamoto, Japan) according to the manufacturer's instructions.

2.5. Western Blot Analyses

Before stimulation, 2 × 10^5 hPASMCs were cultured in serum-starved medium (Humedia-SB containing 1% FBS and antibiotics [Kurabo]) containing the indicated reagents (H-1337, H-1337M1, fasudil, bosentan, or LY294002) for 1 hour, followed by stimulation with serum-starved medium containing 0.1 μM endothelin-1 (Enzo Life Sciences, Farmingdale, NY, USA) or 10 ng/mL recombinant PDGF (PeproTech) for 1 hour. Cellular proteins were then isolated with RIPA buffer (25 mM Tris-HCl [pH 7.6], 150 mM NaCl, 1% NP-40, 1% sodium deoxycholate, and 0.1% sodium dodecyl sulfate) containing protease inhibitors (Protease Inhibitor Cocktail; Nacalai Tesque, Kyoto, Japan) and phosphatase inhibitors (PhosSTOP; Roche, Basel, Switzerland). Proteins from each sample were separated by sodium dodecyl sulfate polyacrylamide gel electrophoresis and transferred onto polyvinylidene difluoride (PVDF) membranes. The membranes were then blocked with 5% bovine serum albumin diluted in Tris-buffered solution containing 0.05% Tween 20 (TBS-T) for 1 hour at room temperature. The blocked membranes were incubated with primary antibodies at 4°C overnight, followed by washing with TBS-T. The membranes were then incubated with a secondary antibody for 1 h at room temperature. Bands were visualized by a chemiluminescence reaction using Pierce ECL Western Blotting (Thermo Fisher Scientific, Waltham, MA, CSA) and LAS-4000 (Fuji Film, Tokyo, Japan). Bands were quantified using ImageJ software program version 1.49 (NIH, Bethesda, MD, USA). Protein expression was normalized to that of β-actin.

2.6. Design of Animal Experiments

Five-week-old male Sprague-Dawley rats weighing 100–140 g were purchased from Japan SLC (Shizuoka, Japan). All rats were housed at 24 °C under a 12-h light/dark cycle in the animal experimental facilities and were kept in autoclaved cages. The animals had free access to drinking water and food. The rats were divided into the following three groups: (1) Su/Hx + H-1337 group, Su/Hx rats administered H-1337; (2) Su/Hx + vehicle group, Su/Hx rats without administration of H-1337; and (3) control group, untreated rats.

2.7. Establishment of the Su/Hx Rat Model

The Su/Hx model was established as described in our previous report [1]. Briefly, SU5416 (20 mg/kg; R&D Systems, Minneapolis, MN, USA), a vascular endothelial growth factor receptor inhibitor, was suspended in carboxyl cellulose buffer containing 0.5% carboxymethylcellulose sodium, 0.9% NaCl, 0.4% polysorbate, and 0.9% benzyl alcohol in deionized water, and the suspension was subcutaneously injected into rats. Less than 30 min after injection, the rats were maintained under normobaric hypoxic conditions (10% O_2) for 3 weeks and subsequently returned to normoxic conditions (21% O_2) for up to 5 weeks.

2.8. Optimization of Treatment Protocols with H-1337 in Su/Hx Rats

From the results of preliminary experiments, the administration route and dose of H-1337 in this study were determined. We found that the H-1337 solution was stable for 7 days at 50 °C (residual rate, 99.9%; Table S2). In a single administration of H-1337 (10 mg/100 g body weight) by sonde, the blood concentration reached a peak (1.47 ± 1.84 μM) in 30 min and decreased to less than half the peak value (0.45 ± 0.46 μM) after 1 h (Figure. 1). This result suggested that it was difficult to maintain a stable blood concentration after a single administration. Continuous administration of the drug in aqueous solution was considered appropriate. When H-1337 was added to the drinking water at concentrations of 0.1, 0.25, or 0.5 mg/mL, the 0.5 mg/mL group showed zero water intake and a weight loss of more than 20% after 48 h, at which point the experiment was terminated for ethical reasons (Figures S1A and S1B). Thus, the maximal concentration of H-1337 that could be given to rats was 0.25 mg/mL.

Based on these preliminary assessments, H-1337 was dissolved in drinking water at 0.25 mg/mL and administered to the Su/Hx + H-1337 group. The Su/Hx + H-1337 group

was continuously treated with H-1337 from day 0 to day 35, and the H-1337 solution was changed every 2 days. Under these conditions, the actual dose of H-1337 was 2–3 mg/day/100 g body weight (Figures S1C and S1D).

2.9. Hemodynamic Measurements

Right heart catheterization was performed at 5 weeks. All rats were placed on controlled heating pads after being anesthetized by an intraperitoneal injection of medetomidine hydrochloride (0.15 mg/kg), midazolam (2 mg/kg), and butorphanol tartrate (2.5 mg/kg). Hemodynamic measurements were performed under normoxic conditions, as previously described, with slight modifications [2]. Polyethylene tube catheters were inserted into the right ventricle (RV) through the right jugular vein to measure the RVSP and carotid artery to measure systolic and diastolic blood pressures. In addition, heart rate was measured. Signals were recorded by PowerLab using the LabChart Pro software program, version 8.1.7 (AD Instrument, Sydney, Australia). After the hemodynamic measurements, the rats were euthanized with pentobarbital sodium (150 mg/kg), and the lungs and hearts were collected for other experiments.

2.10. Assessment of RV Hypertrophy

The extent of RV hypertrophy was assessed based on a previous report [3]. The RV and the left ventricle and septum (LV + S) were weighed. The weight ratio of the RV to LV + S (RV/LV+S) and the ratio of the RV to body weight (RV/BW) were calculated to evaluate the extent of RV hypertrophy.

2.11. Histological and Morphometric Analyses

Resected lung and RV tissues were fixed with 4% paraformaldehyde at 4 °C for at least 48 h. The tissues were embedded in paraffin and sliced to a thickness of 3 μm. The lung sections were deparaffinized and stained with Elastica van Gieson staining. Pulmonary arterial occlusion rates were assessed according to a modified version of the method described in a previous report [4]. All pulmonary arteries with an outer diameter less than 50 μm from one section of each left lobe were counted by investigators blinded to the source of the sections. The arteries were analyzed at 400× magnification using a microscope (Nikon ECLIPSE 55i; Nikon, Tokyo, Japan). The arteries were scored based on the severity of luminal occlusion and the distribution of α-SMA-positive cells, as follows: no evidence of neointimal formation (grade 0), partial (<50%) luminal occlusion (grade 1), and severe (>50%) luminal occlusion (grade 2) according to a previously described method with minor modifications [1,4]. RV myocyte hypertrophy and RV fibrosis were evaluated as previously described, with slight modifications [5,6]. The RV slices were deparaffinized, stained with hematoxylin and eosin (HE) or Masson's trichrome, and observed at 400× magnification using a microscope (Nikon ECLIPSE 55i; Nikon). Cell sizes from HE-stained myocytes were determined by taking the values of minimum diameter, and the area of cardiac fibrosis was scored in a blinded manner in six randomly selected areas per sample. Both evaluations were analyzed using ImageJ software program version 1.49 (NIH). Finally, 3295 arteries from three groups (Su/Hx + H-1337 group, 1255; Su/Hx + vehicle group, 982; and control group, 1058) were histologically assessed.

2.12. Immunohistochemistry

Deparaffinized lung sections were washed and inactivated with endogenous peroxidase with 3% hydrogen peroxide and methanol, followed by blocking with SuperBlock (PBS) Blocking Buffer (Thermo Fisher Scientific, Inc., Waltham, MA, USA) and 0.1% phosphate-buffered saline with TritonX for 30 min at room temperature. The blocked slides were incubated with the primary antibody for 2 h at 25 °C, followed by incubation with a secondary antibody for 30 min at 25 °C. A DAB Substrate Kit (Abcam) was used for detection.

2.13. Immunofluorescence

To evaluate the phosphorylation of each factor, we performed immunofluorescence. Lung and RV specimens were fixed in 4% paraformaldehyde, embedded in optimum cutting temperature compound (Sakura Finetek, Tokyo, Japan), and frozen in liquid nitrogen for tissue sectioning. Ten-micron-thick frozen sections were blocked with SuperBlock (PBS) Blocking Buffer (Thermo Fisher Scientific, Inc.) and 0.1% phosphate-buffered saline with Triton-X for 30 min at room temperature. The following primary and secondary antibodies were used. The sections were observed at 400× magnification and imaged using a fluorescence microscope (Axio Imager A2; Carl Zeiss, Oberkochen, Germany) equipped with AxioVs40×64 V 4.9.1.0 software. The arteries were calculated as the percentage of phospho-MLC-positive or phospho-mTOR-positive tissue with α-SMA-positive cells in a blinded manner in 10 randomly selected areas per sample. In addition, the myocytes were calculated as the percentage of phospho-mTOR-positive troponin T-positive cells in a blinded manner in six randomly selected areas per sample.

2.14. Determination of Blood Concentrations of H-1337 and its Metabolite (H-1337M1) in Rats

Six-week-old male Sprague-Dawley rats were purchased from Japan SLC, Inc. H-1337 was dissolved in 0.3% carboxymethyl cellulose sodium solution at a concentration of 10 mg/mL. Three rats were administered 100 mg/kg H-1337 by oral gavage. Blood samples (200 mL each time) were collected with a small amount of heparin sodium (Mochida Pharmaceutical, Tokyo, Japan) from the tail vein and were centrifuged at 1,000× *g* for 30 min at 4 °C. A plasma sample (70 mL) was collected, and 70 mL of 0.1 M sodium carbonate (pH 9.8) and 350 mL ethyl acetate were added. Samples were then vortexed for 15 min at room temperature and centrifuged at 20,400× *g* for 20 min at 10 °C. The ethyl acetate fraction (300 mL) was collected and evaporated to dryness for 2 h at 40 °C with a vacuum concentrator (SpeedVac; Thermo Fisher Scientific). The residue was dissolved in 140 mL of 0.1% formic acid and centrifuged at 20,400× *g* for 10 min at 10 °C. The supernatant (100 mL) was applied to high-performance liquid chromatography (HPLC) analysis (Shimadzu Co., Kyoto, Japan) for identification and quantification. A Cadenza CD-C18 column (Imtakt, Kyoto, Japan) was incorporated into the HPLC system. The mobile phase consisted of 0.1% formic acid and acetonitrile. H-1337 and H-1337M1 were detected by UV irradiation at a wavelength of 223 nm. HPLC data were analyzed using LC solution software (Shimadzu).

2.15. Metabolism of H-1337 by Rabbit Liver S9 Fraction

A male NZW rabbit liver was homogenized with three volumes of 0.15 M potassium chloride solution. The homogenate was centrifuged twice at 9000 × *g* for 20 minutes at 4 °C. The supernatant was collected as rabbit liver S9 fraction. It (1 mg/mL total protein concentration) was incubated with 50 mM H-1337 in 200 μL of 2% boric acid at 37 °C for one hour. The reaction was stopped by the addition of three volumes of methanol. The sample was incubated at room temperature for 30 minutes and centrifugated at 800 × *g* for 30 min at 20 °C. Subsequently, 720 μL of the supernatant was collected and evaporated to dryness for two hours at 45 °C using a vacuum concentrator (SpeedVac, Thermo Fisher Scientific, MA, U.S.A.). The residue was dissolved in 180 μL of 0.1% formic acid and centrifuged at 20,400 × *g* for 20 minutes at 10 °C. The supernatant was used as a sample for high performance liquid chromatography (HPLC) analysis, and 40 μL of the sample was applied to a HPLC system (Shimadzu Co., Kyoto, Japan). A Cadenza CD-C18 column (Imtakt, Kyoto, Japan) was incorporated into the HPLC system. The mobile phase consisted of 0.1% formic acid and acetonitrile. H-1337 and H-1337M1 were detected by UV at a wavelength of 223 nm. HPLC data was analyzed using LC solution software (Shimadzu).

2.16. Kinase Assays

The kinase activities of ROCK, Akt and MLCK were measured using Eurofines (St. Charles, MO, USA) and SignalChem Biotech Inc. (Richmond, BC, Canada), respectively, according to established protocols.

2.17. Statistical Analyses

Quantitative data are presented as means ± standard deviations unless otherwise stated. Comparisons between two or three or more groups were made using Student's *t*-tests or analysis of variance with Bonferroni's post-hoc test, respectively. Statistical significance was set at $P < 0.05$. Statistical analyses were performed using the GraphPad Prism software program, version 8.0.2 (GraphPad Software, La Jolla, CA, USA).

References

1. Kato, F.; Sakao, S.; Takeuchi, T.; Suzuki, T.; Nishimura, R.; Yasuda, T.; Tanabe, N.; Tatsumi, K. Endothelial Cell-Related Autophagic Pathways in Sugen/hypoxia-exposed Pulmonary Arterial Hypertensive Rats. *Am. J. Physiol. Lung Cell. Mol. Physiol.* **2017,** *313,* L899–L915. DOI: 10.1152/ajplung.00527.2016.
2. Abe, K.; Toba, M.; Alzoubi, A.; Ito, M.; Fagan, K.A.; Cool, C.D.; Voelkel, N.F.; McMurtry, I.F.; Oka, M. Formation of Plexiform Lesions in Experimental Severe Pulmonary Arterial Hypertension. *Circulation* **2010,** *121,* 2747–2754. DOI: 10.1161/CIRCULATIONAHA.109.927681.
3. Fulton, R.M.; Hutchinson, E.C.; Jones, A.M. Ventricular Weight in Cardiac Hypertrophy. *Br. Heart J.* **1952,** *14,* 413–420. DOI: 10.1136/hrt.14.3.413.
4. Toba, M.; Alzoubi, A.; O'Neill, K.D.; Gairhe, S.; Matsumoto, Y.; Oshima, K.; Abe, K.; Oka, M.; McMurtry, I.F. Temporal Hemodynamic and Histological Progression in Sugen5416/hypoxia/normoxia-exposed Pulmonary Arterial Hypertensive Rats. *Am. J. Physiol. Heart Circ. Physiol.* **2014,** *306,* H243–H250. DOI: 10.1152/ajpheart.00728.2013.
5. Fang, Y.H.; Piao, L.; Hong, Z.; Toth, P.T.; Marsboom, G.; Bache-Wiig, P.; Rehman, J.; Archer, S.L. Therapeutic Inhibition of Fatty Acid Oxidation in Right Ventricular Hypertrophy: Exploiting Randle's Cycle. *J. Mol. Med. (Berl.)* **2012,** *90,* 31–43. DOI: 10.1007/s00109-011-0804-9.
6. Pang, H.; Han, B.; Yu, T.; Zong, Z. Effect of Apelin on the Cardiac Hemodynamics in Hypertensive Rats With Heart Failure. *Int. J. Mol. Med.* **2014,** *34,* 756–764. DOI: 10.3892/ijmm.2014.1829.
